# Supplementary material for: Elevated Plasma Levels of C1qTNF1 Protein in Patients with Age-Related Macular Degeneration and Glucose Disturbances
Source: J Clin Med. 2022 Jul 28;11(15):4391. doi: 10.3390/jcm11154391 (PMC9369205; doi:10.3390/jcm11154391)
Supplement: Supplementary file 1 [file jcm-11-04391-s001.zip › jcm-1784722-supplementary.pdf]

Supplementary Table S1. Characteristics and comparison of AMD 0 and AMD 1 groups.

Variables	AMD 0	AMD 1	MD/OR 95% CI	p
	N=279	N=42		
AOC3 (NPX)	4.12 (3.97; 4.38)	4.23 (4.03; 4.44)	-0.11 (-0.28; 0.07)	0.228
ICAM1 (NPX)	7.06 (6.88; 7.30)	7.02 (6.96; 7.32)	0.04 (-0.28; 0.08)	0.252
ICAM3 (NPX)	3.71 (3.55; 3.86)	3.72 (3.59; 3.99)	-0.01 (-0.27; 0.07)	0.255
ICAM-2 (NPX)	5.77 ± 0.29	5.75 ± 0.50	0.02 (-0.25; 0.29)	0.880[1]
NFATC3 (NPX)	1.21 (0.92; 1.83)	1.48 (1.05; 1.80)	-0.17 (-0.38; 0.29)	0.719
ADA (NPX)	5.92 (5.73; 6.13)	6.00 (5.81; 6.35)	-0.08 (-0.36; 0.05)	0.129
ARTN (NPX)	1.24 (1.04; 1.39)	1.21 (1.00; 1.51)	0.03 (-0.20; 0.19)	0.980
AXIN1 (NPX)	2.50 ± 0.49	2.43 ± 0.47	0.07 (-0.19; 0.34)	0.584[1]
Beta-NGF (NPX)	0.43 (0.36; 0.48)	0.43 (0.31; 0.48)	0.00 (-0.04; 0.08)	0.582
CASP-8 (NPX)	2.62 (2.48; 2.84)	2.72 (2.53; 2.98)	-0.10 (-0.27; 0.08)	0.245
CCL11 (NPX)	8.70 ± 0.36	8.87 ± 0.34	-0.17 (-0.36; 0.03)	0.098

CCL19 (NPX)	9.74 (9.44; 10.36)	9.95 (9.43; 10.26)	-0.21 (-0.35; 0.42)	0.929
CCL20 (NPX)	7.37 (6.86; 7.83)	7.50 (7.12; 8.15)	-0.13 (-0.56; 0.37)	0.631
CCL23 (NPX)	10.34 ± 0.46	10.38 ± 0.51	-0.04 (-0.36; 0.24)	0.743
CCL25 (NPX)	6.96 ± 0.46	6.92 ± 0.57	0.04 (-0.21; 0.30)	0.739[1]
CCL28 (NPX)	3.06 ± 0.38	3.18 ± 0.41	-0.12 (-0.36; 0.11)	0.249[1]
CCL3 (NPX)	6.64 (6.43; 6.93)	6.53 (6.30; 6.96)	0.11 (-0.21; 0.31)	0.646
CCL4 (NPX)	7.36 (6.90; 7.64)	7.18 (6.95; 7.63)	0.18 (-0.32; 0.31)	0.901
CD244 (NPX)	6.62 ± 0.30	6.58 ± 0.38	0.04 (-0.14; 0.20)	0.711[1]
CD40 (NPX)	11.85 ± 0.33	11.87 ± 0.24	-0.02 (-0.19; 0.16)	0.828[1]
CD5 (NPX)	5.85 (5.62; 6.04)	5.93 (5.85; 6.07)	-0.08 (-0.27; 0.06)	0.192
CD6 (NPX)	6.76 (6.42; 7.02)	6.76 (6.65; 6.92)	0.00 (-0.32; 0.17)	0.667
CD8A (NPX)	10.14 ± 0.64	10.03 ± 0.61	0.11 (-0.23; 0.46)	0.520[1]

CDCP1 (NPX)	3.47 (3.14; 3.89)	3.84 (3.26; 4.11)	-0.37 (-0.55; 0.15)	0.281
CSF-1 (NPX)	10.63 ± 0.18	10.62 ± 0.19	0.01 (-0.09; 0.11)	0.860[1]
CST5 (NPX)	6.93 ± 0.45	7.07 ± 0.51	-0.14 (-0.39; 0.10)	0.247[1]
CX3CL1 (NPX)	4.99 ± 0.34	5.07 ± 0.31	-0.08 (-0.26; 0.10)	0.394[1]
CXCL10 (NPX)	10.09 (9.61; 10.47)	10.27 (9.77; 10.79)	-0.18 (-0.56; 0.24)	0.335
CXCL11 (NPX)	8.47 (8.01; 8.86)	8.62 (8.27; 8.88)	-0.15 (-0.43; 0.22)	0.524
CXCL1 (NPX)	10.14 ± 0.57	10.13 ± 0.39	0.01 (-0.29; 0.30)	0.947[1]
CXCL5 (NPX)	12.89 (12.31; 13.34)	12.61 (12.30; 12.91)	0.28 (-0.16; 0.55)	0.288
CXCL6 (NPX)	9.52 ± 0.57	9.60 ± 0.69	-0.08 (-0.46; 0.32)	0.702
CXCL9 (NPX)	7.26 (6.90; 7.70)	7.53 (6.90; 7.81)	-0.27 (-0.51; 0.23)	0.466
DNER (NPX)	9.22 ± 0.23	9.24 ± 0.33	-0.02 (-0.20; 0.17)	0.883[1]
EN-RAGE (NPX)	5.32 ± 0.76	5.50 ± 0.63	-0.18 (-0.59; 0.22)	0.361[1]

FGF-19 (NPX)	8.71 ± 0.84	9.15 ±0.88	-0.44 (-0.90; 0.02)	0.060[1]
FGF-21 (NPX)	6.08 ± 1.18	5.87 ± 1.07	0.21 (-0.43; 0.84)	0.527[1]
FGF-23 (NPX)	1.35 (1.18; 1.57)	1.21 (1.12; 1.65)	0.14 (-0.14; 0.25)	0.488
FGF-5 (NPX)	1.65 ± 0.24	1.64 ± 0.26	0.01 (-0.12; 0.14)	0.873[1]
Flt3L (NPX)	9.90 (9.71; 10.19)	9.91 (9.71; 10.05)	-0.01 (-0.19; 0.19)	0.981
GDNF (NPX)	2.33 ± 0.27	2.41 ± 0.35	-0.08 (-0.23; 0.07)	0.293[1]
HGF (NPX)	9.53 (9.37; 9.77)	9.62 (9.32; 9.89)	0.09 (-0.24; 0.19)	0.924
IFN-gamma (NPX)	7.37 (7.01; 7.90)	7.72 (6.78; 8.05)	-0.35 (-0.53; 0.54)	0.935
IL10 (NPX)	4.05 (3.84; 4.30)	3.91 (3.68; 4.16)	0.14 (-0.08; 0.39)	0.166
IL-10RA (NPX)	1.20 (1.06; 1.55)	1.16 (1.02; 1.45)	0.04 (-0.12; 0.21)	0.621
IL-10RB (NPX)	6.68 ± 0.27	6.69 ± 0.21	-0.01 (-0.15; 0.14)	0.955[1]
IL-12B (NPX)	6.70 ± 0.60	6.70 ± 0.55	0.00 (-0.32; 0.32)	0.978[1]

IL13 (NPX)	0.85 (0.66; 1.10)	0.75 (0.59; 0.93)	0.10 (-0.07; 0.27)	0.251
IL-15RA (NPX)	1.92 ± 0.29	1.85 ± 0.28	0.07 (-0.08; 0.23)	0.332[1]
IL-17A (NPX)	2.24 (1.95; 2.65)	2.22 (1.76; 2.47)	0.02 (-0.23; 0.38)	0.625
IL-17C (NPX)	3.43 (2.96; 4.03)	3.16 (2.97; 3.52)	0.27 (-0.20; 0.56)	0.350
IL18 (NPX)	9.42 ± 0.50	9.53 ± 0.51	-0.11 (-0.38; 0.16)	0.424[1]
IL-18R1 (NPX)	8.94 ± 0.38	8.77 ± 0.40	0.17 (-0.04; 0.37)	0.111[1]
IL-1 alpha (NPX)	0.85 (0.70; 0.99)	0.85 (0.62; 1.01)	0.00 (-0.15; 0.19)	0.746
IL-20 (NPX)	0.79 (0.71; 0.89)	0.79 (0.72; 0.91)	0.00 (-0.08; 0.08)	0.929
IL-20RA (NPX)	1.39 (1.25; 1.53)	1.27 (1.16; 1.46)	0.12 (-0.05; 0.24)	0.202
IL-22 RA1 (NPX)	2.31 (2.00; 2.66)	2.23 (1.96; 2.49)	0.08 (-0.18; 0.41)	0.425
IL-24 (NPX)	1.21 (0.94; 1.48)	1.18 (0.88; 1.56)	0.03 (-0.40; 0.24)	0.708
IL2 (NPX)	1.14 ± 0.21	1.03 ± 0.25	0.11 (-0.01; 0.23)	0.074[1]

IL-2RB (NPX)	1.02 (0.88; 1.25)	1.08 (0.94; 1.28)	-0.06 (-0.18; 0.10)	0.662
IL33 (NPX)	0.95 (0.78; 1.08)	0.99 (0.86; 1.09)	-0.04 (-0.15; 0.10)	0.626
IL4 (NPX)	0.43 (0.22; 0.81)	0.39 (0.17; 0.73)	0.04 (-0.14; 0.28)	0.529
IL5 (NPX)	1.13 (0.97; 1.43)	1.18 (0.94; 1.96)	0.05 (-0.40; 0.17)	0.502
IL6 (NPX)	2.68 (2.38; 3.11)	2.63 (2.28; 2.85)	0.05 (-0.16; 0.40)	0.368
IL7 (NPX)	3.91 ± 0.43	3.68 ± 0.45	0.23 (-0.01; 0.46)	0.057[1]
IL8 (NPX)	6.85 ± 0.48	6.84 ± 0.47	-0.01 (-0.24; 0.28)	0.899[1]
LAP TGF-beta-1(NPX)	7.76 ± 0.37	7.59 ± 0.41	0.17 (-0.03; 0.37)	0.096[1]
LIF (NPX)	0.25 (0.16; 0.39)	0.21 (0.10; 0.36)	0.04 (-0.05; 0.15)	0.328
LIF-R (NPX)	4.54 (4.34; 4.70)	4.54 (4.42; 4.78)	0.00 (-0.15; 0.11)	0.817
MCP-1 (NPX)	12.70 (12.44; 12.93)	12.74 (12.63; 12.85)	-0.04 (-0.21; 0.16)	0.746
MCP-2 (NPX)	9.90 (9.55; 10.36)	10.16 (9.97; 10.31)	-0.26 (-0.49; 0.13)	0.278

MCP-3 (NPX)	2.91 ± 0.44	2.89 ± 0.36	0.02 (-0.22; 0.24)	0.913[1]
MCP-4 (NPX)	15.41 ± 0.60	15.44 ± 0.42	-0.03 (-0.34; 0.28)	0.848[1]
MMP-10 (NPX)	9.65 ± 0.53	9.64 ± 0.50	0.01 (-0.28; 0.29)	0.968[1]
MMP-1 (NPX)	15.90 (15.24; 16.25)	15.61 (14.91; 15.99)	0.29 (-0.16; 0.69)	0.202
NRTN (NPX)	0.84 (0.70; 1.01)	0.77 (0.72; 0.93)	0.07 (-0.08; 0.17)	0.432
NT-3 (NPX)	2.18 ± 0.28	2.24 ± 0.32	-0.06 (-0.22; 0.9)	0.428[1]
OPG (NPX)	10.75 ± 0.32	10.74 ± 0.41	0.01 (-0.17; 0.18)	0.913[1]
OSM (NPX)	6.57 (6.21; 7.06)	6.74 (5.93; 7.06)	-0.17 (-0.43; 0.38)	0.812
PD-L1 (NPX)	50.90 (5.73; 6.11)	5.91 (5.68; 6.23)	-0.01 (-0.23; 0.17)	0.856
SCF (NPX)	9.95 (9.71; 10.09)	10.00 (9.70; 10.04)	-0.05 (-0.15; 0.17)	0.817
SIRT2 (NPX)	2.94 (2.69; 3.26)	3.01 (2.72; 3.27)	-0.07 (-0.30; 0.20)	0.657
SLAMF1 (NPX)	3.05 (2.77; 3.29)	3.01 (2.74; 3.35)	0.04 (-0.23; 0.25)	0.946
ST1A1 (NPX)	2.85 ± 0.88	3.23 ± 0.78	-0.38 (-0.85; 0.08)	0.108[1]

STAMBP (NPX)	4.23 (4.01; 4.45)	4.33 (4.05; 4.57)	-0.10 (-0.26; 0.12)	0.506
TGF-alpha (NPX)	4.92 ± 0.58	4.95 ± 0.53	-0.03 (-0.34; 0.28)	0.832[1]
TNFB (NPX)	5.38 ± 0.37	5.38 ± 0.42	0.00 (-0.19; 0.21)	0.930[1]
TNF (NPX)	3.18 (2.94; 3.39)	3.24 (2.97; 3.38)	-0.06 (-0.19; 0.17)	0.912
TNFRSF9 (NPX)	7.30 (7.05; 7.52)	7.27 (7.18; 7.65)	0.03 (-0.31; 0.12)	0.342
TNFSF14 (NPX)	6.27 ± 0.61	6.34 ± 0.54	-0.07 (-0.40; 0.25)	0.649[1]
TRAIL (NPX)	8.53 (8.38; 8.73)	8.51 (8.41; 8.60)	0.02 (-0.08; 0.18)	0.475
TRANCE (NPX)	5.28 ± 0.59	5.25 ± 0.56	0.03 (-0.29; 0.35)	0.839[1]
TSLP (NPX)	1.65 (1.41; 2.03)	1.52 (1.28; 1.95)	0.13 (-0.16; 0.37)	0.415
TWEAK (NPX)	9.79 ± 0.32	9.74 ± 0.34	0.05 (-0.13; 0.22)	0.605[1]
uPA (NPX)	10.02 (9.80; 10.26)	10.06 (9.95; 10.22)	-0.04 (-0.23; 0.10)	0.524

Q1/Q3 – Quartile 1 and 3.
